# The US Postmarketing Surveillance Study of Adult Osteosarcoma and Teriparatide: Study Design and Findings From the First 7 Years

**DOI:** 10.1002/jbmr.1768

**Published:** 2012-09-18

**Authors:** Elizabeth B Andrews, Alicia W Gilsenan, Kirk Midkiff, Beth Sherrill, Yun Wu, Beth H Mann, Daniel Masica

**Affiliations:** 1 RTI Health Solutions, Research Triangle ParkNC, USA; 2 Lilly Research Laboratories, Eli Lilly and CompanyIndianapolis, IN, USA

**Keywords:** OSTEOSARCOMA, EPIDEMIOLOGY, TERIPARATIDE, SURVEILLANCE, PARATHYROID HORMONE (PTH)

## Abstract

The Osteosarcoma Surveillance Study, an ongoing 15-year surveillance study initiated in 2003, is a postmarketing commitment to the United States (US) Food and Drug Administration to evaluate a potential association between teriparatide, rhPTH(1–34), a recombinant human parathyroid hormone analog (self-injectable medication to treat osteoporosis), and development of osteosarcoma in response to a finding from preclinical (animal) studies. Incident cases of primary osteosarcoma diagnosed in adults (aged ≥40 years) on or after January 1, 2003, are identified through population-based state, regional, and comprehensive cancer center registries in the US. Information on possible prior treatment with teriparatide, on demographics, and on risk factors is ascertained by patient or proxy telephone interview after patient consent. Between June 2004 and September 30, 2011, 1448 cases (diagnosed 2003 to 2009) were identified by participating cancer registries (estimated to be 62% of all adult cases in the US for that time period); 549 patients or proxies were interviewed. Interviewed patients were similar to noninterviewed patients with regard to mean age, sex, race, and geographical distribution and tumor type and site of tumor. Mean age of those interviewed was 61 years, 46% were female, 86% were white, and 77% were alive when the case was reported to the study investigators. Data collected in the study provide descriptive information on a large number of adults with osteosarcoma, an uncommon malignant bone tumor. After 7 years of the study, there were no osteosarcoma patients who had a prior history of teriparatide treatment. Thus, approximately halfway through this 15-year study, the study has not detected a pattern indicative of a causal association between teriparatide treatment and osteosarcoma in humans. © 2012 American Society for Bone and Mineral Research.

## Introduction

The Osteosarcoma Surveillance Study was established in 2003 as a postmarketing commitment to the Food and Drug Administration (FDA) for teriparatide, a recombinant human parathyroid hormone (PTH) analog, to evaluate a potential association between teriparatide and osteosarcoma in humans based on preclinical (animal) findings. Teriparatide was first approved in November 2002 in the United States. Teriparatide stimulates new bone formation on trabecular and cortical (periosteal and/or endosteal) bone surfaces by preferential stimulation of osteoblastic activity over osteoclastic activity. It is indicated for treatment of osteoporosis in postmenopausal women at high risk for fracture and to increase their bone mass, to increase bone mass in men with primary or hypogonadal osteoporosis at high risk for fracture, and for the treatment of osteoporosis associated with sustained systemic glucocorticoid therapy in women and men at increased risk for fracture.

In initial preclinical studies in rats administered teriparatide, a dose-dependent increase in the risk of osteosarcoma incidence was observed.^1^ Although subsequent studies demonstrated a “no-effect” dose in rats^2^ and no bone tumors in a long-term study of cynomolgus monkeys,^3^ the US product label contains a warning to physicians and patients about a potential risk of osteosarcoma and to use the product only in the absence of other risk factors for osteosarcoma (e.g., Paget's disease of bone, prior radiation therapy, or children or young adults with open epiphyses) and to limit exposure to a maximum of 2 years.

Osteosarcoma in humans is a primary malignant bone tumor (a sarcoma in which the neoplastic cells produce osseous matrix) that occurs with a bimodal age distribution, with peaks in adolescents and the elderly and slightly higher incidence in males than females.^4^ In US adults, incidence varies with age: 1.7 per million in those aged 25 to 59 years and 4.2 per million for those aged 60 years and older.^5^ Although there is limited information about the etiology of osteosarcoma in adults, it has been observed in association with Paget's disease of the bone and after radiation treatment to the bones.^6^, ^7^ In addition, rare inherited disorders, including Li-Fraumeni syndrome (p53 mutation) and retinoblastoma (pRb loss) are associated with increased rates of osteosarcoma.^8^ Other potential risk factors, including trauma/injury at the tumor site, have been suggested.^7^ One study evaluated the potential role of occupational exposures, including exposure to pesticides.^9^ Fluoride exposure from drinking water as a possible risk factor for osteosarcoma in children and young adults has been extensively studied, but the preponderance of data has not supported a causal association.^10^, ^11^ At present, the majority of osteosarcomas are diagnosed in patients without identified risk factors.^8^

Because teriparatide use is limited and the total number of osteosarcoma cases is uncommon, the cohort and case-control study designs traditionally employed in epidemiologic postapproval safety evaluations were considered inappropriate at the time of the initial study plan to address the research question. Therefore, a surveillance study was designed in which adult cases of osteosarcoma are identified by participating population-based cancer registries and participating medical center cancer registries. Exposure to teriparatide is ascertained through interview and compared with the expected rate of exposure in this population to identify any potential signal of an increased risk of osteosarcoma. This article presents the study methodology and interim results from the first 7 years of this 15-year study. Interim results from a companion osteosarcoma surveillance study that is being conducted in five Nordic countries have been previously published.^12^

## Materials and Methods

### Design

This epidemiologic study identifies osteosarcoma cases in adults from cancer registries in the US. Data on date of osteosarcoma diagnosis, morphology, and topography, and patient contact information are captured from cancer registries; information on drug and environmental exposures, demographics, and brief medical history are collected from patient (or proxy) telephone interview. For a sample of patients each year, patient-reported exposure to osteoporosis medications is verified through chart abstraction. Data are monitored on an ongoing basis for signal detection. A final report is planned at the conclusion of the study.

The study investigators are epidemiologists at RTI International (RTI), an independent, nonprofit research institute. The study is sponsored by Eli Lilly and Company (a pharmaceutical company) with advice and review of interim results by the Osteosarcoma Surveillance Study Advisory Board, composed of members external to RTI and Lilly. Study progress is regularly reported to the US FDA and the European Medicines Agency as well as other regulatory bodies worldwide.

### Case identification

Cases are currently identified through 15 registries—12 state cancer registries, 2 medical center registries, and 1 regional cancer registry—that cumulatively include approximately 62% of all cases of adult osteosarcoma occurring annually in the US. Cancer reporting is mandatory in all states of the US, and registries collect cancer diagnoses for 96% of the US population.^13^ Registries receive reports from physicians, treatment and radiation facilities, hospitals, and pathology laboratories. For this study, we define osteosarcoma cases as histologically confirmed sarcoma that produces osseous matrix and falls within one of the following categories (International Classification of Diseases for Oncology, Third Edition ([ICD-O-3]):

- 9180, Osteosarcoma, NOS
- 9181, Chondroblastic osteosarcoma
- 9182, Fibroblastic osteosarcoma
- 9183, Telangiectatic osteosarcoma
- 9184, Osteosarcoma in Paget's disease of bone
- 9185, Small cell osteosarcoma
- 9186, Central osteosarcoma
- 9187, Intraosseous well-differentiated osteosarcoma
- 9192, Parosteal osteosarcoma
- 9193, Periosteal osteosarcoma
- 9194, High-grade surface osteosarcoma
- 9195, Intracortical osteosarcoma

To conduct a broad-based review of possible bone sarcoma/osteosarcoma cases, data from the following five ICD O-3 morphology codes are also collected where site of the primary cancer was indicated as bone (8800 sarcoma, NOS; 8801 spindle cell sarcoma; 8810 fibrosarcoma, NOS; 8830 malignant fibrous histiocytoma; 9243 dedifferentiated chondrosarcoma). Given the complexities involved in precise diagnostic classification of uncommon sarcomas, the data on cases identified with these five codes are collected in the same manner as the cases of osteosarcoma and evaluated for additional screening. The results are not pooled with the 12 osteosarcoma codes. Summary interim results for these additional five ICD-O codes are included in the Discussion section.

Cancer registries consolidate information obtained from different sources for a single patient, including the first 6 months of treatment, before the registry database is ready for research use. Study patients are identified by cancer registries through regular review of the registry database once it is considered complete or by “rapid case ascertainment” of patients shortly after diagnosis for some registries. The information on patients with osteosarcoma is typically provided to the study investigators 9 to 18 months after the date of reported diagnosis.

### Data collection

Information on potential risk factors for osteosarcoma, drug and environmental exposures, demographics, and other information are collected from telephone interviews with the patient. A proxy familiar with the patient's medical history is interviewed if the patient is deceased or unable to participate.

The procedure for contacting patients and the questionnaire to be used has been approved by a central institutional review board (IRB), as well as local IRBs and other committees affiliated with the cancer registries. Procedures for patient contact are customized to the requirements of each registry. In general, upon identification of an eligible case, the registry provides patient contact and cancer diagnosis information to the study investigators, who contact the physician (when applicable) listed in the registry record to obtain permission to contact the patient or the patient's proxy. In some cases, the local cancer registry is required to contact the physician and/or patient to obtain permission before releasing the information to the investigators. Once permission is obtained to contact the patient, a trained telephone interviewer calls the patient or proxy; provides a brief introduction to the study and invites the patient or proxy to participate; and obtains verbal informed consent before administering a 25- to 30-minute telephone interview. In addition, patients and proxies are requested to provide signed informed consent for the patient's medical records to be reviewed after the interview is complete. Beginning in September 2008, a $25 compensation for time spent was provided to patients or proxies who completed the telephone interview.

The interview includes detailed questions to collect information on teriparatide exposure, including probes for any medication with similar characteristics: The product is stored in the refrigerator and is self-administered as a single daily injection. In addition, the interview ascertains the following information intended to characterize the patients: demographics, including race, age, and state of residence; and a brief medical history, including cancer, osteoporosis, history of medication use, and treatments such as use of other osteoporosis medications. The interview requests data on known risk factors for osteosarcoma: Paget's disease of the bone and radiation treatment and the anatomical site of the radiation treatment. It also requests information that was considered in 2003, at the time of study initiation, to be of interest in exploring the potential etiology of osteosarcoma: history of bone fracture or infection at tumor site; chemotherapy; family history of osteosarcoma and selected other cancers; lifestyle habits such as smoking and alcohol use; and occupational and environmental exposures. No questions were included regarding levels of fluoride ingestion.

Data collection was initiated in July 2004 for patients diagnosed January 1, 2003, or later, and at the conclusion, the study will include incident cases diagnosed through December 31, 2017.

### Analysis

Descriptive analyses are conducted to summarize the main outcomes, including demographic profile, tumor topography and morphology distribution, prevalence of potential risk factors (lifestyle exposures, treatment, injury, infection history, environmental exposures, and personal and family health history). In addition, anatomical sites of prior radiation treatment are compared with the site of the tumor.

Prior teriparatide exposure is derived from the interview data. To place this information into context, we calculate the expected number of osteosarcoma patients who would have received teriparatide if there were no association between drug exposure and disease. This estimate is based on age- and sex-adjusted background rates of osteosarcoma (3.3 per million person-years) and the estimated number of cumulative person-years at risk among patients treated with teriparatide in the geographic regions under surveillance. The estimate is further refined to account for the numbers of osteosarcoma patients identified and interviewed in this study. Using analytic methods common in public health surveillance, we calculate an incidence ratio to compare observed and expected exposure. The 15-year study was designed to detect a doubling of the background rate of osteosarcoma, if it occurs, which would result in 1 additional case per 313,000 treated patients. Details of the planned analyses are contained in the statistical analysis plan for this study.

At least twice a year, the study advisory board reviews the cumulative results from the study and all other available information accumulated by the sponsor to evaluate whether the evidence to date is suggestive of a potential association between teriparatide use and osteosarcoma. The committee also suggests study modifications as needed.

### IRB

The Osteosarcoma Surveillance Study has been approved by the RTI Institutional Review Board (IRB); 4 cancer registries defer to RTI's IRB, and 11 local cancer registry IRBs approved the study.

## Results

**Fig. 1:**
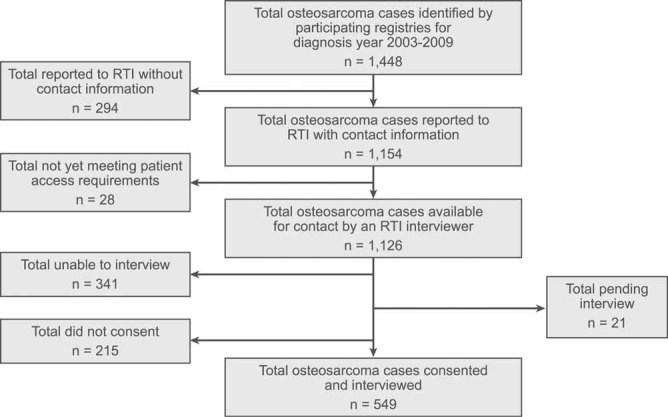
Flow of data collection and attrition of osteosarcoma cases at each step.

As of September 30, 2011, a total of 1448 osteosarcoma cases had been identified by the 15 participating registries for diagnosis years 2003 to 2009. Of those, 1126 have been reported to the investigators with contact information and met all requirements to be interviewed. Of 1126 eligible osteosarcoma patients, 549 (49%) have been interviewed. Of these, 213 (39%) interviews were completed by a proxy rather than by the patient. Of the 577 patients not interviewed, 341 (59%) could not be located or the patient was unable to complete the interview (e.g., owing to illness, hearing impairment) and no proxy was available, and 215 refused to participate in the study (Fig. 1). The refusal rate was higher among proxies (33%) than patients (24%).

**Fig. 2:**
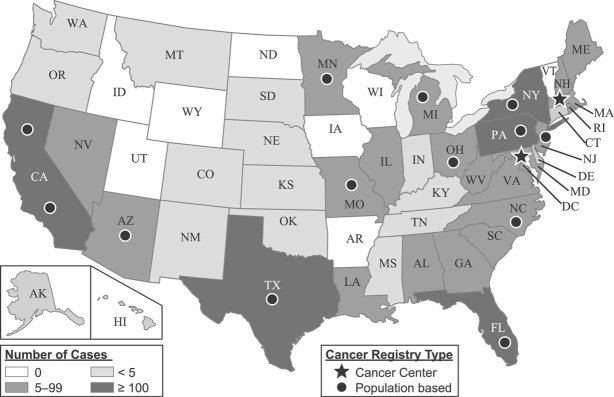
US registries contributing data and residence of cases identified as of September 30, 2011.

Fig. 2 illustrates the distribution of the cases identified by state of residence at the time of diagnosis and the geographic location of participating registries. As expected, the largest number of the cases identified are also from the most populous states (ie, California, Texas, Florida, Pennsylvania, and New York).

### Patient characteristics

The majority of patients interviewed were white (86%). Fifty-four percent were male. As shown in Table 1, patients in the first three 10-year age groups (40 to 49, 50 to 59, and 60 to 69) each constituted approximately one-fourth of the study population. Patients aged 70 or older comprised 27% of the respondents. Mean age was 61 years. At the time the cancer registries reported patients to RTI-HS, 23% of the patients were deceased.

**Table 1:** Demographic and Tumor Characteristics of Respondents and Nonrespondents

Characteristic	Respondents (*n* = 549)	Nonrespondents (*n* = 899)
Age at diagnosis (years)		
40–49	134 (24%)	198 (22%)
50–59	143 (26%)	208 (23%)
60–69	129 (23%)	164 (18%)
70–79	91 (17%)	177 (20%)
80–89	48 (9%)	133 (15%)
≥90	4 (1%)	19 (2%)
Mean (SD)	60.5 (12.8)	63.3 (14.4)
Range	40 to 93	40 to 97
Sex		
Female	251 (46%)	463 (52%)
Male	298 (54%)	435 (48%)
Unknown	0 (0%)	1 (<1%)
Hispanic origin?		
No	394 (72%)	691 (77%)
Yes	25 (5%)	96 (11%)
Unknown	130 (24%)	112 (12%)
Race		
African-American	48 (9%)	121 (13%)
White	471 (86%)	715 (80%)
Other	16 (3%)	34 (4%)
Unknown	14 (3%)	29 (3%)
Vital status		
Deceased	124 (23%)	457 (51%)
Living	422 (77%)	436 (48%)
Unknown	3 (1%)	6 (1%)
ICD-O-3 code		
9180 Osteosarcoma NOS	388 (71%)	599 (67%)
9181 Chondroblastic osteosarcoma	65 (12%)	104 (12%)
9182 Fibroblastic osteosarcoma	38 (7%)	81 (9%)
9183 Telangiectatic osteosarcoma	11 (2%)	20 (2%)
9184 Osteosarcoma in Paget's disease of bone	11 (2%)	42 (5%)
9185 Small cell osteosarcoma	5 (1%)	6 (1%)
9186 Central osteosarcoma	7 (1%)	10 (1%)
9187 Intraosseous well-differentiated osteosarcoma	2 (<1%)	3 (<1%)
9192 Parosteal osteosarcoma	19 (3%)	26 (3%)
9193 Periosteal osteosarcoma	2 (<1%)	6 (1%)
9194 High-grade surface osteosarcoma	1 (<1%)	2 (<1%)
Cancer site category		
Leg bones	170 (31%)	253 (28%)
Pelvis/sacrum/coccyx	87 (16%)	153 (17%)
Skull/face/mandible	80 (15%)	139 (15%)
Scapula/hand/arm bones	50 (9%)	91 (10%)
Connective and soft tissue	58 (11%)	69 (8%)
Ribs/sternum/clavicle	42 (8%)	45 (5%)
Bone and joints (unspecified)	22 (4%)	53 (6%)
Vertebrae	13 (2%)	35 (4%)
Breast	8 (1%)	26 (3%)
Other	17 (3%)	32 (4%)
Unknown	2 (<1%)	3 (<1%)

Source: Cancer registry data.

**Fig. 3:**
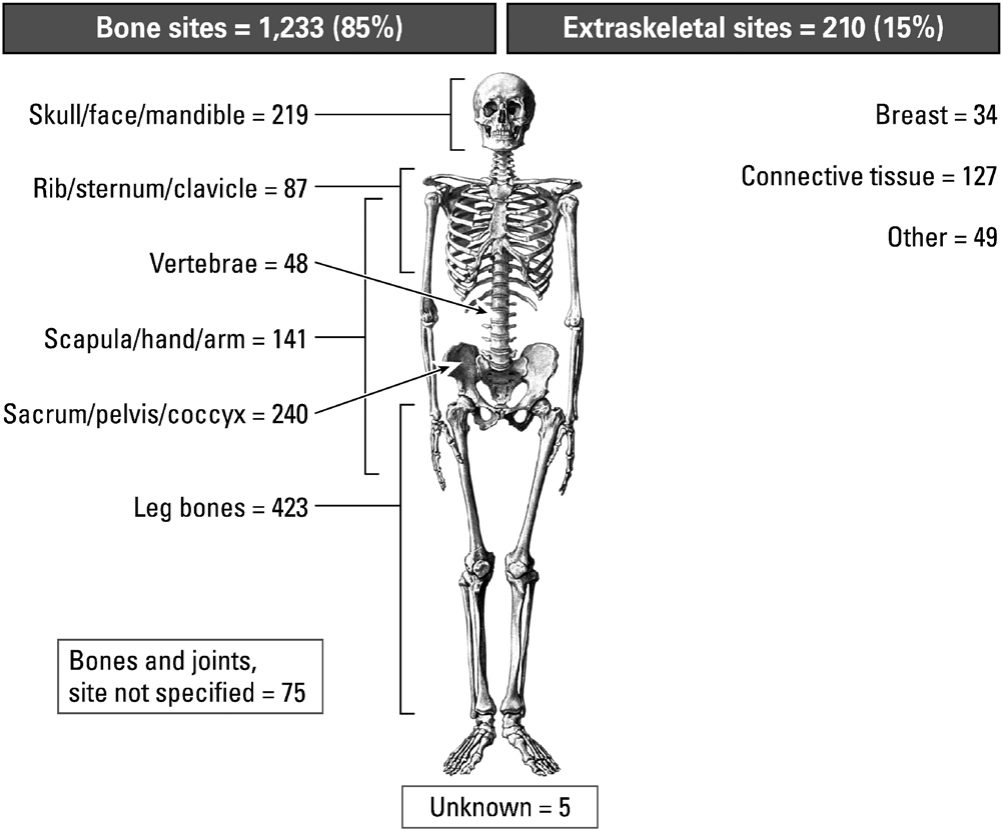
Distribution of sites among all osteosarcoma cases identified by participating cancer registries (*n* = 1448).

Of the 549 interviewed patients, 388 patients (71%) were diagnosed with osteosarcoma NOS, 65 patients (12%) with chondroblastic osteosarcoma, and 38 patients (7%) with fibroblastic osteosarcoma. The other eight morphologic types accounted for the remainder of the diagnoses. The most common site of the primary tumor was in the lower extremities, with 31% occurring in the legs. Another 16% of tumors occurred in the pelvic region, and 15% in the craniofacial bones (Table 1). Tumor site distribution for all cases identified, regardless of interviewed status is shown in Fig. 3.

Table 1 also shows the characteristics of the patients interviewed (respondents) and those who were identified by participating registries but could not be interviewed (nonrespondents). The distributions of patient characteristics are similar between respondents and nonrespondents, with exception of a lower percentage of nonrespondents (48%) than respondents (77%) alive at the time they were reported to RTI by participating registries.

### Medication exposure

Given the number of patients exposed to teriparatide in the US, the background incidence rate of osteosarcoma, the study coverage of osteosarcoma cases, and the interview rate, we expected to find one or two reported teriparatide exposures among patients interviewed to date, in the absence of any association between drug and disease. To date, we have found no valid reports of teriparatide use before diagnosis of osteosarcoma. However, we identified one patient with a preexisting osteosarcoma who had been prescribed teriparatide. In addition, 9% of males and 35% of females self-reported using at least one other medication for osteoporosis at some point in their lifetime. Abstraction of medical records for a 10% random sample of interviewed patients revealed a high concordance between patient-reported and chart-confirmed exposures (≥90%) for these osteoporosis medications.

Although we present data only for patients diagnosed in 2003 to 2009 and interviewed through September 31, 2011, as of submission of this manuscript (June 2012), there have been no new reports from subsequent interviews in this study of teriparatide use before diagnosis of osteosarcoma.

### Patient history and potential risk factors

In 549 interviews, the following known risk factors were reported before the osteosarcoma diagnosis (Table 2): 32 (6%) reported a history of Paget's disease and 107 (19%) had prior radiation treatment. For patients reporting prior radiation treatment, 73% of the cancers developed in the sites or regions of radiation.

**Table 2:** Selected Medical History Among Osteosarcoma Cases (*n* = 549)

Exposure/characteristic	*n* (%)
Known risk factors	
History of Paget's disease of bone	32 (6%)
Prior radiation treatment	107 (19%)
Radiation treatment site matched region/site of tumor	78 of 107 (73%)
Potential risk factors	
Previous injury or infection at tumor site	102 (19%)
Prior chemotherapy treatment	60 (11%)
Family history of osteosarcoma	33 (6%)
Lived within ½ mile of a farm where pesticides could have been used	137 (25%)
Occupational petrochemical exposure	66 (12%)
Worked in job where pesticides were manufactured, mixed, stored, or applied	24 (4%)
Lived within 10 miles of a nuclear power plant	35 (6%)
Occupational radiation exposure	38 (7%)
Other patient characteristics	
Patient history of other cancers	142 (26%)
Family history of breast cancer	125 (23%)
Family history of brain cancer	49 (9%)
Drank alcohol during 12 months before diagnosis	351 (64%)
Smoked at least 100 cigarettes in their lifetime	281 (51%)

Source: Telephone interviews.

We also collected descriptive information on events that have been explored as potential risk factors for osteosarcoma: 60 (11%) reported prior chemotherapy, 102 (19%) reported a history of some kind of injury or infection at the site of the osteosarcoma, and 33 (6%) reported a family history of osteosarcoma. With regard to environmental exposures, 66 (12%) reported having been exposed to petrochemicals in their occupation, 24 (4%) reported workplace exposure to pesticides, and 35 (6%) had lived within 10 miles of a nuclear power or nuclear waste facility.

Additional characteristics reported by patients or their proxy included: family history of breast cancer (23%) and family history of brain cancer (9%). With regard to lifestyle exposures, 281 patients (51%) reported a history of smoking at least 100 cigarettes in their lifetime, and 351 (64%) consumed alcohol in the year before their cancer diagnosis (Table 2).

## Discussion

In this study, we are collecting patient characteristics, medical characteristics, and history of exposures among a large number of adults with osteosarcoma. The 15-year study was designed to detect a doubling of the background rate of osteosarcoma, if it occurs, which would result in one additional case per 313,000 treated patients. Halfway through this study, we have interviewed 549 adults with osteosarcoma and none reported prior exposure to teriparatide. This observation is consistent with the background rate, in the absence of a drug-disease relationship. At this time, we have adequate power to detect a risk, if it occurs, of one additional case per 78,000 treated patients (ie, a fivefold increase in risk), without regard to latency.

In a cancer surveillance study, one must consider the potential latency between the exposure and the appearance of an increase in the number of clinically observed cancer patients. Among known examples of therapeutic exposures associated with cancer (hormones, immunosuppressants, and radiation exposure) the observed latency periods have ranged from less than a year to a decade or longer. Absent a predefined drug-induced model for latency, we assume that any relevant increase in risk, if it exists, would begin to be evident during the 15-year period of this study. Among the more than 16,000 patients who received teriparatide in controlled clinical trials and observational studies in the last 15 years, the largest of which included approximately 4000 patients treated for up to 2 years and followed for an additional 2 years, no cases of osteosarcoma have been reported. In addition, Lilly has maintained a worldwide safety monitoring program for teriparatide, and osteosarcoma surveillance continues to be a major focus. As of June 2012, more than 1 million patients worldwide have received treatment with marketed teriparatide, with approximately 4 million patient-years of cumulative time after initial teriparatide treatment. There have been three published case reports of osteosarcoma in patients who have received marketed teriparatide treatment.^14^–^16^ In addition to these three published reports, there have been a small number of other spontaneous reports of osteosarcoma in the teriparatide-treated population. The cumulative number of spontaneous reports with a pathology-confirmed diagnosis of osteosarcoma does not exceed what would be predicted based on background incidence. In addition, no cases of teriparatide use have been observed in the companion osteosarcoma study being conducted in the Nordic countries, although the number of osteosarcoma cases in that study is small compared with the US study size.^12^

In comparing the results of our study with other evaluations of osteosarcoma in the literature, the average age of this case series is consistent. The race distribution is similar to that reported in the US SEER data for all cancers (84% white) and osteosarcoma (82% white) in persons over 40 years.^17^ The majority of the cases were reported in long bones, but 87 (16%) were reported in the pelvic region and 80 (15%) in the craniofacial bones. As in the case series reported from the companion study in five Nordic countries,^12^ we observed results consistent with those of Unni and Dahlin^7^ and Grimer and colleagues,^6^ who reported an association between radiation site and tumor site. In our case series, 14% of all osteosarcoma patients reported prior radiation therapy at the site corresponding to their tumor, consistent with the finding that 8% of osteosarcomas in patients over the age of 40 years may be associated with prior radiation treatment.^6^ In this study, we frequently observed a reported history of bone fractures, joint replacement, and infection or trauma at the site of the tumor before diagnosis. Without a comparison group, we are not able to draw conclusions about whether these factors or other potential risk factors, such as occupational exposures, are higher than would be observed in a similar group of individuals without osteosarcoma.

In evaluating the findings of the first 7 years of this study, we considered potential biases. As has been seen in other studies involving personal interviews, there are currently many hurdles that diminish investigators' abilities to achieve high response rates. Thus, the focus among survey researchers is changing from maximizing absolute response rates to minimizing potential biases in the responses.^18^ Our ability to interview approximately half of the identified patients reported to RTI with contact information and eligible to be contacted by an RTI interviewer reflected the impact of a long lag time between diagnosis of cancer and the interview attempt (an average of 10 to 27 months for interviews conducted in 2004 to 2009), missing or incomplete current contact information for patients, and refusals from patients and proxies. The study experienced delays at local cancer registries as they began implementation of the HIPAA regulations,^19^ which became effective at the same time this study was initiated. Under the best circumstances, cancer registries tend to have a minimum lag time of 9 to 18 months before data can be released. In our study, the lag time was sometimes longer because we added registries to the study in a sequential manner through 2010, yet attempted to interview patients ascertained through all the registries who were diagnosed as early as 2003. The 49% interview rate might be of greater concern if the nonrespondents differed from respondents in their use of teriparatide. However, we have no reason to suspect that the methods for identifying and recruiting patients for interview could have been biased based on prior medication use. In conducting interview studies, it is always possible that the individuals who chose to participate differed in important ways from those who refused or could not be contacted. We compared characteristics of the respondents and nonrespondents and did not see any patterns suggestive of a bias that could relate to teriparatide use.

It is possible that teriparatide exposure was not accurately reported in the interviews. However, we enhanced the likelihood of eliciting accurate exposure information by including questions about any exposure that might indicate teriparatide use, including probes for indication (osteoporosis), storage requirements (refrigeration), route (self-injection,) and timing of administration (daily), in addition to a question about the specific product name (Forteo). If responses to any of the probes were positive, we considered the patient may have been exposed to teriparatide until we could rule out such exposure by further telephone interview and/or chart review. We believe that recall for use of this particular product was likely to be very high compared with products with less unique features (e.g., oral medications). The fact that we identified a teriparatide exposure that followed the diagnosis of osteosarcoma (see the Medication Exposure section) and that we elicited reports of other osteoporosis medications at a frequency typical for this elderly patient population demonstrated that the study design is effective in identifying relevant medication exposures.

A large percentage of interviews were completed by proxies for patients who were deceased. It is possible that some proxies were not fully aware of patient exposures, which could explain the higher interview refusal rate among proxies than patients. We required proxies to be at least 18 years of age, to be knowledgeable about the patient's medical history, and to report the patient's name and date of birth before the interview proceeded. Most proxies were the spouse or an adult offspring of the patient.

Using state-based cancer registries for case ascertainment provides researchers the opportunity to identify a high percentage of cases of adult osteosarcoma occurring within population-based catchment areas. We estimate that the combination of 12 state-based registries, two medical center registries, and one regional cancer registry identified approximately 60% of all cases of adult osteosarcoma in the US. Because the data are confirmed from multiple sources, including pathology reports, we can be confident that these reports meet an appropriate case definition for osteosarcoma.

To conduct a broad-based review of possible bone sarcoma/osteosarcoma cases (see Methodology), data were also collected from five ICD O-3 morphology codes where the primary sarcoma site was indicated as bone. Among those patients, we have not observed any reported cases of teriparatide exposure.

Because reporting of incident cancer cases is mandatory and because information on any reported case is derived from multiple sources, reporting osteosarcoma cases to cancer registries is unlikely to be influenced by prior exposure to teriparatide. Therefore, we conclude that inaccurate or incomplete reporting from cancer registries is an unlikely source of bias for this study.

The primary objective of this ongoing study is to identify osteosarcoma patients with prior exposure to teriparatide. Based on the first 7 years of this 15-year study, with no exposed patients observed, the study does not support a pattern indicative of a causal association between teriparatide treatment and osteosarcoma in humans. This osteosarcoma surveillance study and others are ongoing to further clarify the potential relationship between treatment and disease, if one exists.

Surveillance studies serve a valuable purpose in helping reduce the amount of uncertainty around possible increased risks of rare events potentially associated with medications. Ideally such a study could be conducted using a single source of preexisting data, such as healthcare claims or electronic medical records linked with cancer registry data at a national level. However, no existing data source is large enough to study an outcome as infrequent as osteosarcoma. Moreover, existing claims data do not contain sufficient clinical detail to distinguish primary osteosarcoma from other tumors located in bone, generally have only a few years of patient follow-up time, and cannot be linked with cancer registries at a national level. Therefore, our study combined existing information from individual cancer registries with primary data collection from patients and proxies to help reduce the uncertainty relating to teriparatide use and osteosarcoma. These surveillance data should be helpful to clinicians and patients as they weigh possible risks against potential benefits of treating osteoporosis patients at high risk for fracture.

## Disclosures

EBA, AWG, KM, BS, and YW are employees of RTI Health Solutions (RTI-HS), a nonprofit research institute that was contracted by Lilly to conduct the study. RTI-HS has responsibility for the design and conduct of the study and the analysis and reporting of the results. The contract between RTI-HS and Lilly assures independent publication rights for RTI-HS. DM and BHM are employees and stockholders of Eli Lilly and Company. The authors have no other financial disclosures to report.
